# Aging in HIV-Infected Subjects: A New Scenario and a New View

**DOI:** 10.1155/2017/5897298

**Published:** 2017-12-21

**Authors:** Eugenia Negredo, David Back, José-Ramón Blanco, Julià Blanco, Kristine M. Erlandson, Maite Garolera, Giovanni Guaraldi, Patrick Mallon, José Moltó, José Antonio Serra, Bonaventura Clotet

**Affiliations:** ^1^Lluita contra la Sida Foundation, Hospital Universitari Germans Trias i Pujol, Universitat Autònoma de Barcelona, Badalona, Spain; ^2^Universitat de Vic-Universidad Central de Catalunya (UVIC-UCC), Vic, Spain; ^3^Department of Molecular and Clinical Pharmacology, University of Liverpool, Liverpool, UK; ^4^Department of Infectious Diseases, Hospital San Pedro-CIBIR, Logroño, Spain; ^5^AIDS Research Institute-IRSICAIXA, Hospital Universitari Germans Trias i Pujol, Universitat Autònoma de Barcelona, Badalona, Spain; ^6^Department of Medicine, University of Colorado, Aurora, CO, USA; ^7^Neuropsychology Unit-Brain, Cognition and Behavior: Clinical Research, Consorci Sanitari de Terrassa, Barcelona, Spain; ^8^Department of Medical and Surgical Sciences for Children & Adults, University of Modena and Reggio Emilia, Modena, Italy; ^9^University College Dublin School of Medicine and Medical Science, Dublin, Ireland; ^10^Geriatric Department, Hospital General Universitario Gregorio Marañón, Instituto de Investigación Sanitaria Gregorio Marañón, Facultad de Medicina, Universidad Complutense, CIBERFES, Madrid, Spain

## Abstract

The prevalence of HIV-infected people aged 50 years or older is increasing rapidly; the proportion will increase from 28% to 73% in 2030. In addition, HIV-infected individuals may be more vulnerable to age-related condition. There is growing evidence that the prevalence of comorbidities and other age-related conditions (geriatric syndromes, functional or neurocognitive/mental problems, polypharmacy, and social difficulties) is higher in the HIV-infected population than in their uninfected counterparts. However, despite the potential impact of this situation on health care, little information exists about the optimal clinical management of older HIV-infected people. Here we examine the age-related conditions in older HIV-infected persons and address clinical management according to author expertise and published literature. Our aim is to advance the debate about the most appropriate management of this population, including less well-studied aspects, such as frequency of screening for psychological/mental and social and functional capabilities.

## 1. Introduction

Our society is aging at an unprecedented rate. People aged 65 and older make up 17% of the population, and by 2030, when the youngest baby boomers turn 65, an estimated 26% of the population could be considered “elderly” [1].

The prevalence of HIV-infected people aged 50 years or older is also increasing rapidly. According to data from the Dutch ATHENA cohort, the proportion of adults aged 50 and older will increase from 28% in 2010 to 73% in 2030 [2]. In addition, HIV-infected individuals may be more vulnerable to age-related conditions [3, 4]. This population often exhibits a higher number of comorbidities and other age-related conditions at a younger age than in the general population [4–9].

Age-associated inflammation, or “inflammaging,” is a major risk factor for both morbidity and mortality in older adults [10]. Chronic inflammation not only impacts the functioning of the immune system, but also contributes to an increased prevalence of many diseases in the general aging process, including cardiovascular disease, diabetes, and cancers. When immune function declines, chronic infections (including CMV and HIV) may be reactivated, stimulate the innate immune system, and trigger a subclinical inflammatory response that, in turn, stimulates adaptive immune responses, thus creating a vicious circle. Among HIV-infected individuals, the presence of subclinical chronic inflammation related to HIV infection is also associated with major age-related complications [11] and has been linked to a decreased survival among these patients [12].

Despite the heterogeneous nature of the aging process, common health characteristics of older adults include multiple comorbidities, geriatric syndromes (falls, incontinence, frailty, dementia, confusion, malnutrition, sarcopenia, and disability), polypharmacy, social difficulties (e.g., isolation, poverty, and lack of caregivers), and atypical clinical presentations of common health conditions (e.g., depression without sadness, infection without fever or leukocytosis, and acute coronary or acute abdominal syndrome without pain). Older patients often experience more than one geriatric syndrome or manifestations of aging, as there is a large overlap between the aetiological factors.

Despite the potential impact of this situation on health care, the optimal clinical management of older HIV-infected people is not well defined. We examine the age-related conditions in older HIV-infected persons and provide suggestions on clinical management according to published literature and guidelines [13], as well as the expertise of the authors. Our aim is to advance the debate about the most appropriate management of older HIV-infected individuals.

The observations we make are not focused on specific comorbidities, which have received considerable attention and may have separate published guidelines. Rather, our objective is to address integration of various guidelines in addition to less well-studied aspects.

## 2. Clinical Conditions

With the aging of the HIV-infected population, the burden of noncommunicable diseases or HIV-associated non-AIDS conditions has been increasing steadily [3–9, 14]. From 2003 to 2013, the US Medicaid Database reported an increasing prevalence of CVD (3% to 7%), renal impairment (5% to 11%), osteoporosis (4% to 6%), and diabetes mellitus (9% to 19%) [15]. As a result, non-AIDS-related mortality has eclipsed AIDS-related mortality as the major cause of death in HIV-infected persons with widespread access to antiretroviral therapy [16–18].

In addition, there is growing evidence that the prevalence of these comorbidities and other age-related conditions (functional or neurocognitive/mental problems) is higher in the HIV-infected population than in their uninfected counterparts [19].

### 2.1. Comorbidities

The increased prevalence of CVD in this group reflects the complex interaction between age-related factors of this population: the high prevalence of classic cardiovascular risk factors (tobacco use, dyslipidaemia, diabetes, recreational drug use, etc.), the inflammatory status, and the effects of HIV replication and antiretroviral therapy [20, 21]. HIV infection itself, as well as the impact of HIV on gut permeability, resulting in bacterial translocation, may lead to the development of accelerated atherosclerosis and decreased high-density lipoprotein (HDL) levels [22]. Additionally, proinflammatory populations of T cells and activated monocytes may lead to functional or structural vascular changes linked with the development of coronary plaques [23].

Guidelines for the screening of CVD in the setting of HIV infection largely follow recommendations for the general population [24]. However, both the Framingham risk score and the 2013 American College of Cardiology/American Heart Association (ACC/AHA) guidelines have been shown to underestimate the CVD risk in this population [25]. Current recommendations to reduce CVD risk are mainly focused on the viral suppression and the appropriate management of traditional risk factors. Special emphasis is placed on cigarette smoking; the high prevalence of smokers among the HIV-infected population urges the inclusion of smoking treatment protocols in current models of HIV care. Future and ongoing studies such as the REPRIEVE study (the use of pitavastatin to reduce the risk of CVD in HIV-infected adults) will inform future decisions about how to manage cardiovascular risk factors and reduce systemic inflammation (e.g., using statins).

Chronic kidney disease (CKD) is also more common in patients with chronic HIV infection than in the general population [26]. CKD accounts for significant morbidity and mortality in 30% of HIV-infected individuals [27]. Other common risk factors include increasing age, hyperlipidaemia, diabetes, exposure to some antiretroviral drugs, and hepatitis B and hepatitis C. Concomitant bone disease is also an emerging comorbidity. The prevalence of osteopenia and osteoporosis is increased in HIV-infected persons (up to 60% and 10–15%, resp.), although the mechanism and consequences of these changes are not fully understood [28]. Screening and assessment of bone disease is thoroughly described in the European AIDS Clinical Society (EACS) guidelines [29] and other guidelines for the management of bone disease [30].

As the incidence of cancer increases exponentially with age, it is expected that the number of patients with concurrent malignancies will increase. Indeed, HIV infection predisposes to several types of cancer, in particular those associated with chronic viral infections such as Epstein-Barr virus (non-Hodgkin lymphoma), human herpes virus 8 (Kaposi's sarcoma), hepatitis B and C viruses (hepatocellular carcinoma), and human papillomavirus (cervical intraepithelial neoplasia and anal intraepithelial neoplasia) [31, 32]. At present, there are no specific data regarding whether the clinical presentation is altered in HIV-infected patients or whether management of cancer in HIV-infected aging patients should differ from standard screening protocols.

### 2.2. Neurocognitive Impairment

HIV-associated neurocognitive disorder (HAND) covers a spectrum of neurocognitive impairment, from asymptomatic neurocognitive impairment and mild neurocognitive disorder to HIV-associated dementia (HAD). HAND includes a variety of cognitive symptoms, such as problems in attention/working memory, learning/memory, executive function, and processing speed.

There is consistent evidence that neurocognitive ability is commonly impaired in HIV-infected patients; about half of all antiretroviral-treated patients present with cognitive impairment. Advanced age increases susceptibility to HAND in HIV-infected individuals, leading to alteration of the presentation of HAND and an increase in the proportion of older HIV-infected adults with HAD. Although greater neuropsychological impairment affects older HIV-infected persons more frequently than younger infected individuals, many middle-aged HIV-infected individuals are also experiencing cognitive decline similar to that found in much older non-HIV-infected adults [33].

Direct damage caused by the virus, as well as indirect factors (e.g., increased risk of CVD, chronic drug use/abuse, and potentially toxic long-term antiretroviral use), has a synergistic negative effect on the central nervous system [34]. Persistent inflammatory response and neuroimmunological disorders, vascular and metabolic comorbidities, and neurotoxicity have been identified as potential causes of neural injury in the older HIV-infected population. Common neuroimaging findings in HIV-infected persons, particularly among older adults, include brain atrophy (cortical and subcortical), elevations in abnormal white matter signal on magnetic resonance, metabolic abnormalities, and variations in regional glucose metabolism. These trends in neuropsychological and neuroimaging data point to the significance of an interaction of HIV with the aging brain. However, it is still unknown whether this interaction directly affects neurodegenerative processes, accelerates normal cognitive aging, or contributes to other comorbidities affecting the brain in older adults [33].

Of particular concern for researchers, clinicians, and patients is the decline in everyday functioning (e.g., instrumental activities) that is observed in a significant proportion of adults with HIV and is clearly more prominent among older adults. Older HIV-infected patients are particularly vulnerable to decline in everyday functioning, mainly in instrumental activities. These findings are not surprising, because neuropsychological functioning is predictive of impairment in everyday functioning. Cognitive impairment may be detrimental to the real-world functioning of older HIV-infected adults and could potentially interfere in basic activities such as antiretroviral adherence or ability to drive.

Assuming the clinical relevance and public health impact of neuropsychological impairment on older HIV-infected adults, there is a growing need to identify factors associated with successful cognitive aging (e.g., cognitive reserve and lifestyle).

**Figure 1:**
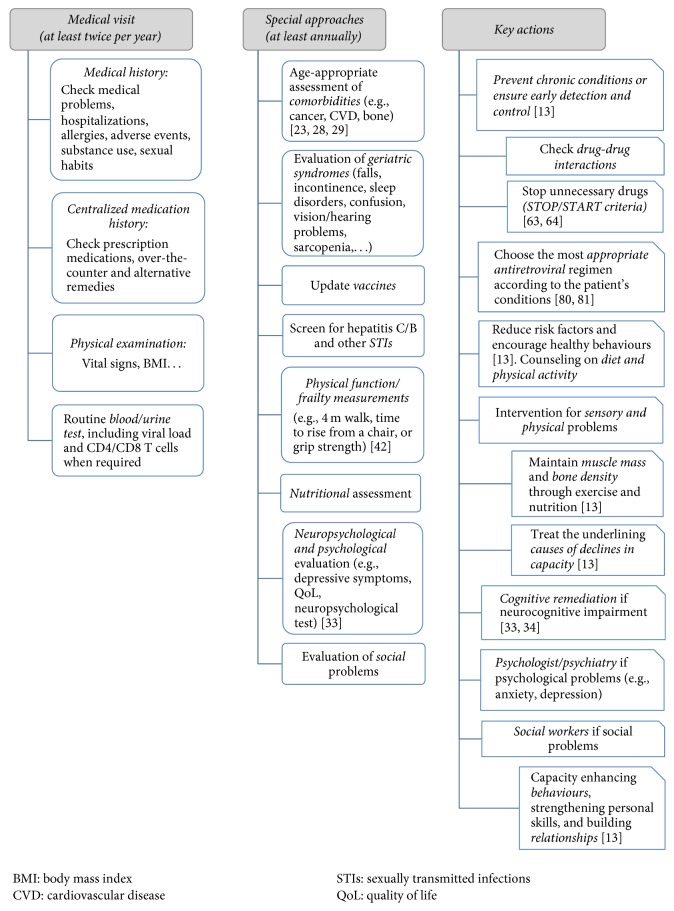
*General and specific considerations for older HIV-infected subjects*. CVD, cardiovascular disease; STIs,* sexually transmitted infections*; QoL, quality of life.

In addition, a systematic and appropriate neuropsychological evaluation of older adults with HIV should be incorporated into clinical practice to identify patients with cognitive impairment, predict their functional capacity, and determine the best potential therapeutic approach for delaying or reducing the impact of cognitive impairment [34]. The age-related cognitive decline among HIV-uninfected subjects can be partially reduced by mental training and physical activity [35] (Figure 1). Similar considerations should be given to treating age-associated cognitive decline in HIV-infected persons.

### 2.3. Functional Limitations and Frailty

In the geriatrics literature, the well-described frailty phenotype is regarded as a loss of functional homeostasis that leaves a person unable to effectively recover from various stressors and is associated with poor health outcomes, including excess mortality [36]. Ultimately, the cumulative burden of comorbidities and their respective therapies, as described above, may lead to frailty in HIV-infected persons. Indeed, based on a variety of definitions, the prevalence of frailty or a frailty-like syndrome has been reported in 5–19% of HIV-infected cohorts [37]. Initial studies among HIV-infected populations describe the higher prevalence of frailty in persons with the deepest immune compromise, and subsequent studies have continued to demonstrate an association between HIV serostatus and frailty, regardless of effective antiretroviral therapy [38–42]. In addition to the syndrome of frailty, impairment in objective measures of physical function is also apparent. Compared with demographically similar non-HIV-infected adults, HIV-infected persons appear to experience early impaired physical function [43] that ranges from more subtle impairments at a high level of functioning, such as lower peak exercise capacity [44], to more severe impairments such as difficulty completing a quarter-mile walk or a short physical performance battery [45–47].

The clinical consequences of these impairments in physical function and frailty are readily apparent: impaired physical function and frailty have been associated with increased risk of insulin resistance [48], falls [49], impaired quality of life [50], and hospitalizations [45, 51]. Furthermore, the cooccurrence of both HIV infection and impaired physical function or frailty has a synergistic effect: in the ALIVE cohort, predicted mortality was significantly greater with the combination of both HIV and impaired physical function than with HIV infection or impaired physical function alone [46]. Similarly, the impact of both HIV infection and frailty also had a synergistic effect on mortality [52].

In the HIV-uninfected population, only a small proportion of frail individuals will return spontaneously to full robustness [53]. Interventions aimed at increasing physical activity and improving nutrition have been shown to be effective [54–56]. While evaluation of physical function and frailty is not routinely assessed in most HIV clinics, simple measures such as gait speed on 4-meter walk, time to rise from a chair, and grip strength could provide clinically relevant data [43, 57] (Figure 1). Repeat measurements can advance understanding of the underlying causes (e.g., HIV, age, and comorbidities) and guide interventions to prevent, slow, or reverse impairments and frailty.

## 3. Polypharmacy and Drug-Drug Interactions

The development of comorbidities in older HIV-infected patients often results in initiation of new treatments, in addition to ongoing antiretroviral treatment. Consequently, older HIV-infected patients tend to use a higher number of concomitant medications, especially cardiovascular therapies, central nervous system agents, and medications to treat gastrointestinal disorders [58]. For example, 14% of patients aged more than 65 years in the Swiss HIV Cohort Study were taking 4 or more concomitant medications [59].

The term “polypharmacy” is used to describe consumption of multiple (usually considered more than 5), excessive, unnecessary, or nonindicated drugs [60]. Complex treatment containing a high number of drugs is associated with a greater likelihood of medication errors and lack of adherence [61]. In addition, polypharmacy may cause adverse effects, particularly in older patients with underlying comorbidities and altered drug metabolism [62, 63]. Finally, concomitant use of multiple drugs may result in drug-drug interactions, which can further impact treatment safety, efficacy, and adherence [58]. According to Marzolini et al. [58], nearly half of HIV-infected people aged 50 or older may be receiving concomitant drugs that are potentially prone to significant interactions with antiretroviral therapy, especially when the antiretroviral regimen included boosted antiretroviral drugs.

Interactions with antiretroviral drugs are driven mainly by induction or inhibition of enzymes and transporters involved in drug disposition [60]. Ritonavir and cobicistat are both potent inhibitors of the enzyme CYP3A4 and of drug transporters in the gut and liver. Consequently, use of boosted protease or integrase inhibitors may be associated with increased exposure to a large number of concomitant agents (e.g., hypnotics, antidepressants, lipid-lowering agents, antiarrhythmics, and corticosteroids), eventually leading to a high risk of adverse effects. Conversely, efavirenz and etravirine can induce the activity of enzymes and transporters, leading to subtherapeutic concentrations of other drugs in some cases. Finally, the unboosted integrase inhibitors raltegravir and dolutegravir seem to be associated with a lower risk of interactions with other drugs.

Antiretroviral drugs may be both perpetrators and victims of interactions if they are combined with other enzyme inducers (e.g., rifampin, older antiepileptics) or inhibitors (e.g., antifungals). Additionally, the absorption of some antiretrovirals may be altered by other medications through chelation of divalent cations (e.g., integrase inhibitors) and through changes in gastric pH (e.g., atazanavir, rilpivirine).

Management of polypharmacy in the older people can be complex, especially when multiple prescribers are involved in patient care [60]. A centralized medication history is mandatory and should include not only prescription medications but also over-the-counter and alternative remedies (e.g., herbals, supplements) (Figure 1). A critical review of the therapeutic regimen should be made at every patient visit, and unnecessary drugs should be stopped (Beers and STOPP/START criteria) [64, 65]. Finally, specific information on drug-drug interactions should be consulted (Figure 1). Ideally, sources of information on drug-drug interactions should be widely accessible, provide accurate and easy-to-interpret information about the clinical management of these interactions, and undergo regular updates (excellent examples include https://www.hiv-druginteractions.org/ and http://app.hivclinic.ca/). Providers should maintain a proactive approach towards drug-drug interactions and anticipate potential drug-drug interactions both when prescribing a new drug and when deciding to discontinue a drug.

## 4. Clinical Considerations for the Care of Older HIV-Infected Adults

General care of older HIV-infected adults should be based both upon HIV-related conditions and upon other conditions specifically related to the older adult.

### 4.1. Antiretroviral Treatment

The specific antiretroviral regimens for older adults are not different from those recommended for the general HIV-infected population. However, the choice of regimen and potential adverse reactions or drug-drug interactions should be evaluated carefully based on the presence of comorbidities and respective treatments.

Tenofovir disoproxil fumarate (TDF) has been associated with an increased risk of chronic kidney disease [66] and higher prevalence of osteopenia and osteoporosis [67]. The recently approved agent tenofovir alafenamide (TAF) appears to be associated with less kidney disease and less reduction in bone mineral density [68].

Some antiretroviral regimens have been associated with an increase in cardiovascular risk. For example, in the D:A:D cohort study, a significantly increased risk of CVD was detected in patients exposed to indinavir, lopinavir-ritonavir, didanosine, and abacavir [69] and more recently to darunavir [70]. Subsequent studies on the potential association between abacavir and CVD are contradictory [71, 72] and randomized clinical trials [73] and meta-analyses on this topic have not confirmed this association [74, 75]. However, many guidelines recommend caution using abacavir based therapies in patients with underlying cardiovascular disease if other options are available [35, 76].

Because of possible nucleoside/nucleotide reverse transcriptase inhibitor- (NRTI-) related toxicities and drug-drug interactions, especially in older persons, new therapeutic regimens are being investigated. These include dual class therapy with protease inhibitors and integrase inhibitors [77] or lamivudine [78] and NRTI- and protease inhibitor-sparing regimens (e.g., dolutegravir-based dual therapy) [79, 80]. Although the treatments appear promising in initial trials with younger populations, long-term durability in older adults has not yet been established. Concomitant medications (calcium, iron, vitamins) may interfere with the absorption of integrase inhibitors; therefore, careful attention is imperative with these regimens.

Based on the presence of comorbidities, polypharmacy, and pharmacokinetic and pharmacodynamic alterations associated with age, Guaraldi [81] further expanded considerations in the choice of antiretroviral drug for these patients, with an emphasis on integrase inhibitors [82], owing to their tolerability, low toxicity, and low number of drug-drug interactions. However, given that multiple treatment options are available, treatment should be prescribed on an individual basis, informed by underlying resistance, comorbidities, and patient and provider preference.

Finally, until recently, HIV-infected patients over 50 years of age have been poorly represented in clinical trials; consequently, complications and toxicities may be underreported in this collective [83]. Clinical and pharmacokinetic trials with HIV-infected persons aged more than 50 years are essential [84–86]; ongoing trials will provide us with invaluable data.

### 4.2. General Considerations: Next Steps

The needs of an aging population are emphasized in the World Health Organization definition of health: “Health is a state of complete physical, mental and social well-being and not merely the absence of disease or infirmity.” In older adults, the medical, physical, mental, and social aspects of health could be assessed using a comprehensive geriatric assessment (CGA) to ascertain health status and individual needs [87, 88]. CGA is a multidimensional, interdisciplinary diagnostic process that makes it possible to determine the medical, psychological, and functional capabilities of an older person by using specific criteria including age, medical comorbidities, psychosocial problems, previous or predicted high health care utilization, change in living situation, and specific geriatric conditions. It is mainly focused on determining the pathological risk, residual skills, short- and long-term prognosis, and the personalized therapeutic and care plan of the functionally compromised and frail older subjects. A systematic review of data coming from the last three decades about the systematic implementation of CGA programs evidences that mainly complex older subjects may benefit from a CGA, by improving quality of care and reducing hospitalization events [88].

Among HIV-infected persons aged 50 years or older, both general and HIV-specific management considerations can be taken into account and CGA programs should be considered to be incorporated in the HIV clinics in the following years [89, 90]. Figure 1 summarizes some considerations. Of note, these multiple considerations for the older adult with HIV infection should be individualized and prioritized dependent on the care goals of the individual patient.

In summary, aging is a complex process that is more complicated in combination with a chronic disease, such as HIV infection. The geriatric literature suggests that care of the older patient with multimorbidity is best managed with the assistance of a multidisciplinary team. HIV medical care providers caring for older HIV-infected patient with multimorbidity can be guided by these principles of geriatric medicine. Providers should seek to understand the future health needs of older HIV-infected patients and modify the goals of care to meet these needs.
